# Severe Rhabdomyolysis With Stage 3 Acute Kidney Injury After Ayahuasca Use Managed Without Renal Replacement Therapy: A Case Report

**DOI:** 10.7759/cureus.92568

**Published:** 2025-09-17

**Authors:** Arya Kermanshah, Margo Stapleton, Christine M Mauriello, Miguel Cobas, Ivet T Cordoba Torres

**Affiliations:** 1 Medical Education, University of Miami, Miami, USA; 2 Anesthesiology, University of Miami Miller School of Medicine, Jackson Memorial Hospital, Miami, USA; 3 Critical Care Medicine, University of Miami Miller School of Medicine, Jackson Memorial Hospital, Miami, USA

**Keywords:** acute kidney injury, ayahuasca, case report, dimethyltryptamine, nephrotoxicity, rhabdomyolysis

## Abstract

Ayahuasca, a brew containing N,N-dimethyltryptamine (DMT) with monoamine oxidase-inhibiting β-carbolines, has expanded from traditional use to global retreats. Reported adverse effects include vomiting, agitation, serotonin toxicity-like syndromes, and cardiovascular events. Rhabdomyolysis has been described with other classic hallucinogens (e.g., lysergic acid diethylamide and psilocybin), but, to our knowledge, an ayahuasca-associated rhabdomyolysis case has not been previously reported.

Our case is of a previously healthy man who ingested ayahuasca and undertook prolonged travel before presenting with diffuse myalgias and dark urine. Initial laboratory results showed creatine kinase (CK) at 110,659 IU/L, serum creatinine (Cr) at 6.8 mg/dL, and blood urea nitrogen (BUN) at approximately 95 mg/dL. Comprehensive hospital toxicology screens were negative for non-prescribed substances. Despite early treatment, Cr rose to 13.6 mg/dL (hospital day 7), with BUN peaking at approximately 130 mg/dL, consistent with severe acute kidney injury (AKI). CK declined rapidly with care, reaching <1,500 IU/L by day 9 and 730 IU/L by day 11. There was no clinical evidence of compartment syndrome. The patient received guideline-concordant management with high-volume isotonic fluids, electrolyte monitoring and correction, avoidance of nephrotoxins, and Intensive Care Unit (ICU) observation. Kidney function began to downtrend after the Cr peak but remained impaired at discharge, with nephrology follow-up arranged.

This case highlights a plausible multifactorial pathogenesis: catecholaminergic surge and hyperactivity from a serotonergic psychedelic, combined with dehydration and questionable circumstances, culminating in profound rhabdomyolysis and AKI. Recognizing plant-based hallucinogen exposure is critical when evaluating unexplained CK levels that are more than five times the upper limit of normal. Our patient’s trajectory - CK normalization preceding delayed renal recovery - mirrors classic myoglobinuric AKI. Clinicians should consider ayahuasca and other serotonergic hallucinogens when assessing rhabdomyolysis, anticipate renal complications, and institute early, aggressive fluid resuscitation and nephrotoxin avoidance. While rhabdomyolysis is recognized with other hallucinogens, this case suggests ayahuasca may carry similar or synergistic effects.

## Introduction

Several drugs are known to cause rhabdomyolysis, including statins, antipsychotics, antiepileptics, and certain illicit drugs, including cocaine, amphetamines, and heroin. The pathophysiology involves direct myocyte injury or metabolic disturbances, leading to increased intracellular calcium and subsequent muscle cell necrosis [1]. The diagnosis is typically confirmed by elevated serum creatine kinase (CK) levels, often more than five times the upper limit of normal [2]. Importantly, medications and illicit drugs can independently cause rhabdomyolysis without the need for prolonged immobilization or the "found-down" aspect, as evidenced by the diverse range of medications and substances that can induce this condition through various mechanisms.

While substances like cocaine and statins are frequently linked to rhabdomyolysis, less is known about ayahuasca, a psychoactive brew containing N,N-dimethyltryptamine (DMT) and β-carboline alkaloids. Traditional ayahuasca typically combines *Banisteriopsis caapi* (β-carbolines: harmine/harmaline) with a DMT-containing leaf, such as *Psychotria viridis*; β-carbolines inhibit monoamine oxidase A (MAO-A), enabling oral DMT bioavailability [3,4]. Ayahuasca induces altered states of consciousness and has shown promise in treating mental health disorders, such as substance use disorders, anxiety, depression, and post-traumatic stress disorder (PTSD) [5]. Pharmacologically, DMT and β-carbolines interact with serotonergic receptors, potentially offering therapeutic benefits for depression, anxiety, and PTSD [3]. However, its use is associated with risks, including hallucinations, seizures, and death, highlighting the need for caution despite some promising results for mental health [6]. While ayahuasca has historical and modern therapeutic applications, more rigorous clinical trials are needed to assess its safety and efficacy for conditions like depression, anxiety, and PTSD [3]. Ayahuasca's potential to cause rhabdomyolysis is not well documented, which warrants further exploration.

It is well established that it is crucial to avoid nephrotoxic drugs, such as nonsteroidal anti-inflammatory drugs (NSAIDs) and certain antibiotics, which can further exacerbate renal injury [7]. Management strategies for rhabdomyolysis and extremely elevated CK levels primarily focus on early and aggressive fluid resuscitation to prevent acute kidney injury (AKI). The cornerstone of treatment is aggressive intravenous hydration, typically with isotonic saline, aiming for a urine output (UO) of 1-3 mL/kg/h (up to ~300 mL/h), to dilute myoglobin (weight-based target) [7-9]. The use of sodium bicarbonate (NaHCO_3_) for urine alkalinization remains controversial and is not universally recommended, given limited evidence of benefit, though it may be considered in select cases to reduce myoglobin precipitation in the renal tubules [7].

In severe cases, renal replacement therapy (RRT) may be necessary, particularly when there is refractory hyperkalemia, metabolic acidosis, or fluid overload. However, the decision to initiate RRT is not always straightforward. Research, including a Cochrane review and studies by de Fallois et al., suggests that RRT may not improve mortality rates compared to conservative management in patients with rhabdomyolysis [7,10,11]. Moreover, the decision to begin RRT should be guided by clinical indications, such as worsening renal function and electrolyte imbalances, rather than solely by elevated myoglobin or CK levels [12]. Recent meta-analyses similarly show no survival benefit to “early” RRT initiation in AKI without urgent indications; RRT remains indication-driven [13,14].

This report complies with the Health Insurance Portability and Accountability Act (HIPAA); HIPAA authorization for publication was obtained from the patient. Institutional Review Board (IRB) approval was not required for this ≤3-patient case report; however, written consent for publication was obtained.

## Case presentation

A 38-year-old male with no significant past medical or family history presented with generalized weakness and muscle pain after an ayahuasca retreat. The patient ingested ayahuasca approximately six to eight hours before the onset of generalized weakness and muscle pain, and ~24 hours before presentation to our Emergency Department (ED). He consumed an estimated volume of 250-300 mL over three doses, prepared in a traditional retreat setting. He denied co-ingestants, including selective serotonin reuptake inhibitors (SSRIs), statins, stimulants, or other prescribed medications. He reported no exertion, heat exposure, or prolonged immobilization, but described limited hydration during travel, averaging ~500 mL of fluid intake over >12 hours. He was reportedly assaulted repeatedly with thin branches during the retreat, receiving initial care overseas and noted to have minor abrasions on his face, arms, and legs. On his journey home to the U.S., he was brought to our ED via emergency medical services (EMS) during a layover due to worsening symptoms of weakness, fatigue, abdominal pain, and back pain. He arrived afebrile and hemodynamically stable, with severe rhabdomyolysis (CK 110,569 U/L), AKI (initial creatinine (Cr) 6.8 mg/dL), hyperkalemia, hyponatremia, elevated transaminases, and leukocytosis (Table 1). Comprehensive urine and serum toxicology screens were negative for non-prescribed substances, as determined by routine immunoassays (note that DMT/β-carbolines are not included in standard panels). He was admitted to the Intensive Care Unit (ICU) for close monitoring and serial labs. 

**Table 1:** Serial Laboratory Trends by Hospital Day Serial laboratory trends for sodium, potassium, transaminases (AST, ALT), and WBC count over the hospitalization. Values are reported by hospital day (HD 1 = admission). Reference ranges are provided in parentheses. Arrows (↑/↓) denote values above or below the reference range. The table demonstrates persistent hyponatremia, variable hyperkalemia, elevated transaminases, consistent with rhabdomyolysis-associated hepatocellular injury, and leukocytosis that improved with supportive care. AST, Aspartate Aminotransferase; ALT, Alanine Aminotransferase; WBC, White Blood Cell

Hospital Day	Sodium (135-145 mmol/L)	Potassium (3.5-5.0 mmol/L)	AST (0-40 U/L)	ALT (0-40 U/L)	WBC (4-11 × 10³/µL)
HD 1	133 ↓	5.3 ↑	95 ↑	102 ↑	15.7 ↑
HD 2	128 ↓	4.2	97 ↑	101 ↑	10.4-10.7
HD 3	126 ↓	3.6	99 ↑	90 ↓	10.3
HD 4	126 ↓	3.8-5.2	-	-	11
HD 5	123 ↓	4	99 ↑	91 ↓	10.7-11.6 ↑
HD 6	122 ↓	5.3 ↑	108 ↑	93 ↓	10.5
HD 7	125 ↓	4.8	101 ↑	90 ↓	10.4
HD 8	122-124 ↓	4.5-5.0	108 ↑	92 ↓	11.5 ↑
HD 9	127 ↓	4.4	115 ↑	90 ↓	12.0 ↑
HD 10	131 ↓	4.3	128 ↑	87 ↓	16.4 ↑
HD 11	134	3.9	132 ↑	88 ↓	13.7 ↑

A comprehensive trauma evaluation was negative for non-superficial injury, apart from a minimally displaced nasal bone fracture; serial reassessments revealed no evolving traumatic complications. Although the patient reported repeated assault, physical examination and imaging revealed only superficial abrasions and the minimally displaced nasal bone fracture. No deep tissue injury, muscle contusion, or compartment syndrome was present. Comprehensive trauma evaluation and serial examinations excluded crush syndrome as a cause of rhabdomyolysis. He was admitted to the ICU for early, aggressive crystalloids and individualized urine alkalinization (Lactated Ringer's (LR) 100 mL/h plus NaHCO_3_ infusion), treatment of hyperkalemia (insulin/dextrose, calcium), and strict intake/output monitoring with a UO target of ~1-3 mL/kg/h. Admission venous blood gas revealed pH 7.27, pCO_2_ 34 mmHg, and HCO_3_^-^ 15 mmol/L, consistent with metabolic acidosis with partial respiratory compensation. Renal ultrasound showed increased cortical echogenicity without obstruction; serial chest radiographs (CXRs) remained clear.

By ICU day 2, CK began falling, while Cr rose to 8.8 mg/dL. On day 3, a loop-plus-thiazide diuretic approach (bumetanide with a single metolazone dose) was started for net-even volume management after resuscitation, with ongoing balanced fluids and bicarbonate. Diuretics were introduced only after initial resuscitation, once the patient was non-oliguric, and were used solely for volume control to prevent fluid overload, not as a treatment for the rising Cr itself. This supportive strategy aligns with standard non-oliguric AKI management, where the goal is to optimize hemodynamics and avoid pulmonary congestion. In addition, judicious diuresis facilitated careful correction of hyponatremia by promoting greater free-water than sodium excretion, helping stabilize electrolytes while maintaining euvolemia.

**Figure 1:**
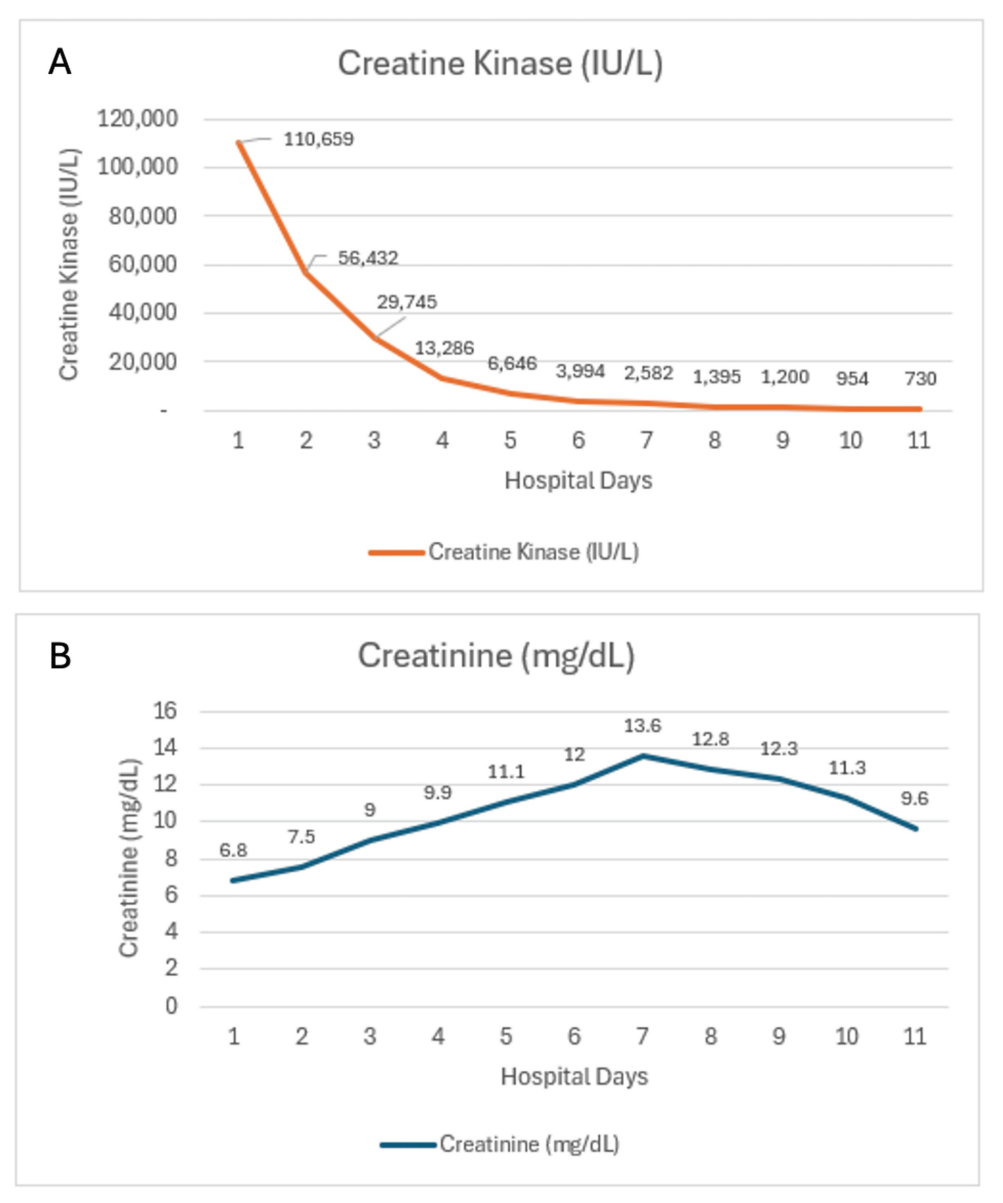
Key Laboratory Trends in Rhabdomyolysis-Associated Acute Kidney Injury Serial laboratory trends during hospitalization for rhabdomyolysis with acute kidney injury. (A) Creatine kinase declined from a peak of 110,659 IU/L on hospital day 1 to 730 IU/L by day 11, representing a >99% reduction. (B) Serum creatinine rose from 6.8 mg/dL on hospital day 1 to a peak of 13.6 mg/dL on day 7, before improving to 9.6 mg/dL by day 11.

On days 4-6, fluids were transitioned to balanced crystalloids; bumetanide was briefly infused at higher rates, and a single dose of acetazolamide was used to mitigate alkalosis during diuresis. Trauma imaging and examinations remained unchanged, with no occult injuries identified. See Figure 1 for CK and serum Cr trend over his hospital course.

By day 7, Cr peaked at 13.6 mg/dL as CK continued to downtrend and the patient entered a polyuric recovery phase (UO ~4.1 L/day), permitting diuretic taper. Days 8-9 remained polyuric (UO ~3.4-3.9 L/day), with gradual fluid reduction (brief use of ½ normal saline (NS) during recovery to match hypotonic urinary losses). By days 10-11, euvolemia was achieved, CK declined to 730 U/L, Cr improved to 9.6 mg/dL, all IV fluids and diuretics were stopped, and the patient was discharged with renal-sparing counseling and nephrology follow-up (30/90-day labs). Despite severe azotemia, predefined RRT triggers - refractory hyperkalemia or acidosis, diuretic-refractory volume overload, or uremic complications - were never met.

The patient was contacted by telephone approximately 30 days and 1 year after discharge and reported complete resolution of myalgias, dark urine, and generalized weakness, with a return to baseline activity and no emergency visits or readmissions. He denied further ayahuasca or other substance use and reported adherence to hydration and avoidance of nephrotoxins (e.g., NSAIDs). At ~90 days, he reportedly remained asymptomatic, without uremic symptoms, and had not required dialysis or repeat hospitalization.

## Discussion

This case of severe rhabdomyolysis and kidney injury involved the combined effects of ayahuasca ingestion and a questionable degree of trauma. The sympathomimetic effects of ayahuasca, which can increase sympathetic tone and systemic vasoconstriction, may further impair renal perfusion and may have contributed to the severity of rhabdomyolysis and acute renal failure in this case. Because this is a single‐patient report, we cannot prove that ayahuasca alone triggered rhabdomyolysis - particularly given concurrent trauma, even if mild - and the absence of three- or six-month post-discharge Cr trends limits our ability to assess long-term renal sequelae. To strengthen causality, we applied the Naranjo Adverse Drug Reaction Probability Scale [15]. The calculated score was 6, indicating a probable relationship between ayahuasca ingestion and the rhabdomyolysis. Key contributors included a plausible temporal association (+2), absence of alternative causes (+2), prior reports of similar hallucinogen toxicity (+1), and objective confirmation with laboratory data (+1). While trauma was initially a concern, the absence of crush injury on imaging and physical examination reduces trauma as a confounder and strengthens the association with ayahuasca.

The case described is unique in the literature, and the association of ayahuasca with severe rhabdomyolysis and AKI appears to be unprecedented - especially when compared to the well-established risks with cocaine and statins - in a patient with negative toxicology and no other medications. To our knowledge, no prior case reports have linked ayahuasca ingestion directly to rhabdomyolysis. Similar hallucinogens (e.g., 3,4-methylenedioxymethamphetamine, or “MDMA”) have been reported to cause rhabdomyolysis, but the pharmacology differs. Unlike cocaine- or statin-induced rhabdomyolysis, where direct myotoxic and mitochondrial dysfunction pathways are well characterized, ayahuasca’s serotonergic and sympathomimetic effects may drive muscle ischemia via different vasoconstrictive and mitochondrial stress mechanisms, suggesting a distinct pathophysiology. The decision by nephrology and critical care to pursue conservative management in this patient is noteworthy and provides valuable guidance - especially for smaller centers without access to specialty services.

The natural history of AKI follows the phases of initiation, extension, maintenance, and recovery. During the extension and maintenance phases, renal function can continue to worsen, as indicated by rising serum Cr levels. This worsening reflects the initial insult and ongoing injury processes, such as inflammation and cellular damage [16,17]. The patient's non-oliguric status after a diuretic challenge with clear UO can be somewhat reassuring, as non-oliguric AKI is often associated with a better prognosis compared to oliguric AKI. However, it does not guarantee recovery, and renal function can still deteriorate [16,18], further exemplifying the complexity of managing a case like this. Our patient was received in the extension-maintenance stage; if identified correctly, management at this stage is primarily supportive. This includes optimizing hemodynamics, avoiding nephrotoxic agents, and correcting electrolyte imbalances. Fluid strategy adhered to guideline targets (UO: 1-3 mL/kg/h, up to 300 mL/h), favoring balanced crystalloids during early resuscitation. Alkaline therapy (NaHCO_3_) was individualized and not used routinely, consistent with contemporary American Association for the Surgery of Trauma and Eastern Association for the Surgery of Trauma (AAST/EAST) guidance [9,19]. Fluid management should be carefully balanced to avoid both hypovolemia and fluid overload. Assess fluid status via clinical exam (turgor, orthostatics, jugular venous pressure), basic labs (blood urea nitrogen to Cr (BUN/Cr) ratio, hematocrit), and ultrasound when available [7]. After an initial bolus of 20-30 mL/kg, further boluses should be guided by dynamic indices (passive leg raise, stroke volume responsiveness). Fluid resuscitation, along with the judicious use of diuretics (such as bumetanide and metolazone), helped manage the patient’s fluid balance and contributed to renal recovery without the need for RRT. RRT may be necessary if there are indications such as refractory hyperkalemia, severe acidosis, or volume overload. The Kidney Disease: Improving Global Outcomes (KDIGO) guidelines emphasize the importance of supportive care and avoiding further insults to the kidneys during the maintenance phase of AKI [20]. 

It is reasonable to assume that, at other centers, the ICU team may have initiated RRT earlier and pursued a more aggressive renal support strategy. In the absence of a dedicated nephrology team, we would have pursued the same aggressive approach. This case serves as a compelling example of the complexities involved in critical care management, particularly when dealing with unusual causes of rhabdomyolysis, such as ayahuasca use, alongside a concerning presentation to the ED. The patient's successful recovery - despite severe biochemical derangements and without the need for RRT - underscores the importance of personalized care. It also provides valuable insights into the decision-making process that may guide similar cases in the future.

With the increasing popularity of recreational ayahuasca use, public health initiatives and regulation are essential to mitigate potential risks, such as rhabdomyolysis and AKI. Further research is essential to define the optimal management strategies for ayahuasca-induced rhabdomyolysis and its long-term effects on renal function and overall health.

## Conclusions

This case illustrates that even severe rhabdomyolysis with stage 3 AKI injury can, in select patients, improve without dialysis when structured supportive care is applied. While a single report cannot establish causality or generalize outcomes, it highlights the importance of early aggressive fluids, electrolyte correction, and nephrotoxin avoidance as potentially sufficient in carefully monitored cases. Despite a CK level above 110,000 IU/L and a Cr peak of 13.6 mg/dL, renal function improved with high-volume isotonic fluids, targeted urine alkalinization, meticulous electrolyte correction, strict avoidance of nephrotoxins, and diuretic-guided volume control while preserving UO. Trauma imaging was reassuring, and hospital toxicology was negative for nonprescribed substances; routine panels do not detect DMT or beta-carbolines, making ayahuasca exposure the most plausible precipitant, likely amplified by dehydration and immobilization. The clinical course followed classic pigment nephropathy, with rapid CK clearance and slower Cr recovery, reinforcing that dialysis should be reserved for established indications, such as refractory hyperkalemia, severe acidosis, diuretic-refractory volume overload, or uremic complications, rather than enzyme thresholds alone. As ayahuasca use becomes more common, clinicians should ask about it explicitly, anticipate rhabdomyolysis and kidney injury, and apply protocolized conservative management that can avert dialysis even in very severe presentations.

## References

[1] Zimmerman JL, Shen MC (2013). Rhabdomyolysis. Chest.

[2] Nance JR, Mammen AL (2015). Diagnostic evaluation of rhabdomyolysis. Muscle Nerve.

[3] Brito-da-Costa AM, Dias-da-Silva D, Gomes NG, Dinis-Oliveira RJ, Madureira-Carvalho Á (2020). Toxicokinetics and toxicodynamics of ayahuasca alkaloids N, N-dimethyltryptamine (DMT), harmine, harmaline and tetrahydroharmine: clinical and forensic impact. Pharmaceuticals (Basel).

[4] Cata-Preta EG, Serra YA, Moreira-Junior ED (2018). Ayahuasca and its DMT- and β-carbolines - containing ingredients block the expression of ethanol-induced conditioned place preference in mice: role of the treatment environment. Front Pharmacol.

[5] Domínguez-Clavé E, Soler J, Elices M (2016). Ayahuasca: pharmacology, neuroscience and therapeutic potential. Brain Res Bull.

[6] Houle SK, Evans D, Carter CA, Schlagenhauf P (2021). Ayahuasca and the traveller: a scoping review of risks and possible benefits. Travel Med Infect Dis.

[7] Long B, Koyfman A, Gottlieb M (2019). An evidence-based narrative review of the emergency department evaluation and management of rhabdomyolysis. Am J Emerg Med.

[8] Coccolini F, Moore EE, Kluger Y (2019). Kidney and uro-trauma: WSES-AAST guidelines. World J Emerg Surg.

[9] Kodadek L, Carmichael Ii SP, Seshadri A (2022). Rhabdomyolysis: an American Association for the Surgery of Trauma Critical Care Committee Clinical Consensus Document. Trauma Surg Acute Care Open.

[10] Zeng X, Zhang L, Wu T, Fu P (2014). Continuous renal replacement therapy (CRRT) for rhabdomyolysis. Cochrane Database Syst Rev.

[11] de Fallois J, Scharm R, Lindner TH, Scharf C, Petros S, Weidhase L (2024). Kidney replacement and conservative therapies in rhabdomyolysis: a retrospective analysis. BMC Nephrol.

[12] Petejova N, Martinek A (2014). Acute kidney injury due to rhabdomyolysis and renal replacement therapy: a critical review. Crit Care.

[13] Castro I, Relvas M, Gameiro J, Lopes JA, Monteiro-Soares M, Coentrão L (2022). The impact of early versus late initiation of renal replacement therapy in critically ill patients with acute kidney injury on mortality and clinical outcomes: a meta-analysis. Clin Kidney J.

[14] Li JH, Cai JH, Wang MJ (2023). Early strategy vs. late initiation of renal replacement therapy in adult patients with acute kidney injury: an updated systematic review and meta-analysis of randomized controlled trials. Eur Rev Med Pharmacol Sci.

[15] Naranjo CA, Busto U, Sellers EM (1981). A method for estimating the probability of adverse drug reactions. Clin Pharmacol Ther.

[16] Bellomo R, Kellum JA, Ronco C (2012). Acute kidney injury. Lancet.

[17] Basile DP, Anderson MD, Sutton TA (2012). Pathophysiology of acute kidney injury. Compr Physiol.

[18] Ronco C, Bellomo R, Kellum JA (2019). Acute kidney injury. Lancet.

[19] Sawhney JS, Kasotakis G, Goldenberg A (2022). Management of rhabdomyolysis: a practice management guideline from the Eastern Association for the Surgery of Trauma. Am J Surg.

[20] Sparrow HG, Swan JT, Moore LW, Gaber AO, Suki WN (2019). Disparate outcomes observed within kidney disease: improving global outcomes (KDIGO) acute kidney injury stage 1. Kidney Int.

